# Clinical pharmacists and drug-related problem management in a South African hospital

**DOI:** 10.4102/hsag.v31i0.3337

**Published:** 2026-02-26

**Authors:** Mukonazwothe J. Luvhimbi, Phumzile Skosana, Nkhensani Shirindza, Elmien Bronkhorst

**Affiliations:** 1 Department of Clinical Pharmacy, School of Pharmacy, Sefako Makgatho Health Sciences University, Pretoria, South Africa

**Keywords:** drug-related problems, clinical pharmacy interventions, pharmaceutical care, rational use

## Abstract

**Background:**

Drug-related problems (DRPs) negatively impact health outcomes and are more likely in patients with multiple comorbidities and polypharmacy. Involving clinical pharmacists in multidisciplinary teams can improve drug safety and efficacy by preventing DRPs.

**Aim:**

This study aimed to identify and analyse DRPs and highlight the role of clinical pharmacists in minimising DRPs.

**Setting:**

This study was conducted in the internal medicine wards of a tertiary hospital in South Africa.

**Methods:**

A descriptive quantitative study was conducted using purposive sampling, including all inpatients in internal medicine wards. Drug-related problems were identified through daily reviews of patient files using a standardised pharmaceutical care form. Findings were communicated to the healthcare team. Data were analysed descriptively using Stata.

**Results:**

A total of 181 patient cases were reviewed, with most patients being male (65.2%) and aged 20–40 years (35.9%). A total of 241 DRPs were recorded, averaging 1.76 DRPs per patient. The most frequent DRP was failure to receive therapy (53.1%), often because of omitted doses. The clinical pharmacist made 277 recommendations, with 37.2% accepted by nurses and 22.7% by doctors.

**Conclusion:**

Clinical pharmacists effectively identified and addressed DRPs, particularly omitted doses and therapeutic duplication. The integration of clinical pharmacists into healthcare teams can significantly reduce DRPs and enhance patient safety.

**Contribution:**

This study raises awareness of drug-related problems and supports the development of strategies to prevent them in hospitalised patients.

## Introduction

Drug-related problems (DRPs) encompass situations in which drug therapy may result in unintended or harmful outcomes including adverse effects, drug–drug interactions, cognitive impairment and metabolic problems (Garin et al. 2021). The risk for these negative health outcomes increases with the number of drugs also known as polypharmacy, which is defined as the concurrent use of five or more drugs (Garin et al. 2021). Together with an increase in life expectancy, polypharmacy has been found to reach up to 90% in adults over 75 years of age (Garin et al. 2021). During hospital admission, adding new medicines for acute health problems may exacerbate the risk of developing DRPs, which can result in potential life-threatening consequences for patients, increased hospital stay and healthcare costs (Abunahlah et al. 2018; Garin et al. 2021; Hoel, Connolly & Takahashi 2021).

The World Health Organization (WHO) has cited DRPs as the leading cause of death in the United States (Ni et al. 2021; WHO 2016). The prevalence of DRPs proves to be a global challenge, as several studies identified DRPs from various countries globally, including those in Africa. The most identified DRPs globally are adverse drug events (ADE) and drug–drug interactions as indicated by Garin et al. (2021), Abunahlah et al. (2018), Annor, Schellack and Gous (2014), Abdel-Latif and Abdel-Wahab (2015), Lenssen et al. (2016) and Sungsana et al. (2023).

Drug-related problems can be defined as any event that occurs during drug therapy, for any indication that can alter the health outcomes or result in undesired outcomes for the patient (Abunahlah et al. 2018; Zaal et al. 2013). The Pharmaceutical Care Network Europe Association (PCNE) developed a classification system to categorise DRPs into four categories, such as Indication, Effectiveness, Drug safety and Drug use (Zaal et al. 2013). The examples of these categories are patients experiencing pain and not receiving pain medication or receiving medicine dosages that are too high [causing toxicity] or too low [causing inefficacy] (Zaal et al. 2013).

Contributing factors to DRPs can stem from errors made during prescribing, dispensing or the administration of medicine. Errors such as the prescriber selecting the wrong medicine or dose, the pharmacist dispensing the wrong medicine or wrong strength or the medicine being administered at the wrong frequency can cause DRPs (Abunahlah et al. 2018; Pharmaceutical Care Network Europe 2019; Pokkuluri et al. 2023). These factors could be prevented by the involvement of the clinical pharmacist as part of the multidisciplinary team, promoting rational drug use and enhancing drug safety and effectiveness (Ahmed et al. 2021; Chevalier et al. 2016; Movva et al. 2015; Pokkuluri et al. 2023). Clinical pharmacists are specially trained professionals who apply knowledge of pharmacology, toxicology, pharmacokinetics and pharmacotherapeutic to optimise drug therapy and provide comprehensive pharmaceutical care, ultimately improving patients’ quality of life (American College of Clinical Pharmacy 2016; Bronkhorst, Schellack & Gous 2022).

The incorporation of clinical pharmacists into multidisciplinary teams has been shown to increase the detection of DRPs according to research (Albayrak & Özbalcı 2024). Interventions described in research studies focusing on DRPs are varied and cover a broad range of aspects, such as medication reconciliation, medication adherence, dose adjustment or therapeutic indication (Garin et al. 2021). Recent international clinical pharmacy forums, including the 50th ESCP symposium, further highlight that managing polypharmacy and ensuring medication safety require highly individualised, interprofessional and person-centred approaches (Dreischulte et al. 2022b).

Clinical pharmacists contribute directly to such approaches through their pharmacotherapy expertise to support multidisciplinary healthcare teams, working to improve how medicines are used in order to meet both patient-centred and broader public health objectives (Dreischulte et al. 2022a).

Clinical pharmacists, through application of pharmaceutical care, can ensure rational drug use by choosing the most appropriate medication, dose and frequency, to ensure cost-effective therapy that ensures safe and effective use of medication, to minimise DRPs (Albayrak & Özbalcı 2024; Bronkhorst et al. 2022; Chauhan et al. 2018; Harikrishna 2021; Mateti et al. 2014).

## Aim of this study

Through the involvement of a clinical pharmacist in the multidisciplinary healthcare team, the study aimed to identify and analyse DRPs. As a result of the identification of DRPs, the study then aimed to explore and describe the role that clinical pharmacists play in healthcare teams to minimise DRPs.

## Research methods and design

### Study design and setting

A descriptive quantitative study design was followed. This study was conducted in the internal medicine wards of a tertiary hospital in South Africa including all in-patients. Patients who were admitted to internal medicine wards for a period of more than 24 h, for a period of 5 months (January 2024–May 2024), were included.

### Study participants and recruitment

The study population included patients who were admitted to internal medicine wards for longer than 24 h. A purposive sampling method was used to include all in-patients in the internal medicine wards, regardless of their diagnosis to reach the study sample. The sample size was calculated using EPI Info™ (v 7.2.5.0), from the bed capacity of the internal medicine wards (132 beds), with a 5% margin of error and a 95% confidence interval. The required sample size for the study was *n* = 124.

### Data collection

The researcher used standardised, validated pharmaceutical care form, developed by the American Society of Hospital Pharmacists, adapted for use in the South African context and utilised in previous studies to collect data from patient files (Bronkhorst et al. 2012; Schellack 2008; Untiedt 2003). Patient records were reviewed daily, and information on vital signs, laboratory results, including microbiological data and medication use were collected. The clinical pharmacist identified DRPs through application of pharmaceutical care and communicated potential or actual problems to the doctors and nurses. The outcomes of the recommendations were observed.

### Data analysis

Data were collected in Excel, cleaned and checked for accuracy. Descriptive analysis was conducted using Stata (v18). The relationship between DRP frequency and factors such as comorbidities, medication count and drug type was analysed using logic regression. Results were presented in tables and graphs. Pearson correlation and Chi-squared tests assessed Cramer’s V, degrees of freedom, Chi-square value and *p*-values. Probability values were calculated to examine correlations between DRPs and influencing factors, including patients’ age, number of comorbidities and prescribed medications. Statistical significance was defined as *p* < 0.05.

### Reliability and validity

To ensure reliability and validity, the pharmaceutical care form was utilised, which has a long history from its development by the American Society of Hospital Pharmacists to its utilisation by the School of Pharmacy and other studies (Bronkhorst et al. 2012; Schellack 2008; Untiedt 2003). Additionally, expert opinions have helped to refine it over the years to ensure its validity (Bronkhorst et al. 2012; Schellack 2008; Untiedt 2003). The pharmaceutical care form was the tool used to collect data, which contains 27 questions on the checklist, to help guide the researcher on what patient information should be collected. This assisted to minimise gaps in the data and ensured the validity of the data. The data was cross checked by the researcher and supervisors to ensure the reliability of the data and results thereof.

### Ethical considerations

Approval was granted by the Ethics Committee of Sefako Makgatho Health Science University (07 September 2023/SMUREC/P/352/2023: PG). Permission was sought from the hospital manager before the study commenced. The study was approved by the National Health Research Database (07 September 2024/GP 202308 071). As this study was a patient record review, no informed consent was required. This study was performed in line with the principles of the Declaration of Helsinki.

## Results

### Demographic and socio-demographic data

During the data collection period, a total of *n* = 181 patient cases were reviewed. Most patients were male (118; 65.2%), aged between 20 and 40 years of age (65; 35.9%), with a median of 44 years (Interquartile Range [IQR] of 29).

From the *n* = 181 patient file reviews, at least one DRP was identified in 137 patients (75.7%). The number of comorbidities identified was 228, with most patients presenting with more than one comorbidity. The most prevalent comorbidities were hypertension (62; 27.2%), HIV/AIDS (53; 23.2%) and diabetes (28; 12.3%). Polypharmacy (five or more medicines per prescription) was identified in 112 (61.9%) of patients, with 80 of them experiencing a DRP, although no statistical significance was observed (*p*-value = 0.089). Table 1 represents the demographic data of the study participants, in relation to DRPs.

**TABLE 1:** Demographic data in relation to drug-related problems.

Characteristic	Total (*n* = 181 patients)		With DRPs		Without DRPs		*p* -value
	*n*	%	*n*	%	*n*	%	
Gender							0.022
Male	118	65.2	83	70.3	35	29.7	-
Female	63	34.8	54	85.7	9	14.3	-
Age groups (years)							0.036
< 20	11	6.1	7	63.6	4	36.4	-
20–40	65	35.9	43	66.2	22	33.8	-
41–60	60	33.1	47	78.3	13	21.7	-
> 60	45	24.9	40	88.9	5	11.1	-
Prescribing patterns							0.089
< 5 drugs	69	38.1	57	82.6	12	17.4	-
5 or more drugs	112	61.9	80	71.4	32	28.6	-

DRPs, drug-related problems.

### Classification of drug-related problems

A total of 241 DRPs were identified from the *n* = 181 patient file reviews, with a mean incidence of 1.76 DRP per patient. Failure to receive therapy (128; 53.1%) was the most identified DRP, with missed doses being the highest contributor. Almost half of the identified DRPs (122; 50.6%) were the result of administration errors, and 119 DRPs (49.4%) were because of prescribing errors.

Table 2 provides an overview of the classification of the identified DRPs.

**TABLE 2:** Breakdown of the drug-related problems according to pharmaceutical care network Europe Association classification.

Drug-related problems	Frequency (*N* = 241)	
	*n*	%
**Failure to receive therapy**	**128**	**53.1**
Missed doses	112	46.5
Drug indicated but not administered	10	4.1
Prescribed drug out of stock	6	2.5
**Drug regimen problem**	**43**	**17.8**
Dose timing instructions wrong, unclear or missing	14	5.8
Drug dose too high	11	4.5
Route of administration omitted	9	3.7
Drug dose too low	7	2.9
Inappropriate dosage form	2	0.8
**Therapeutic duplication**	**16**	**6.6**
Duplication of active ingredient	16	6.6
**Inappropriate therapy length**	**16**	**6.6**
Duration not indicated	15	6.2
Duration of treatment too long	1	0.4
**Drug interaction**	**14**	**5.8**
Drug–drug interactions	14	5.8
**Inappropriate drug selection**	**13**	**5.4**
Inappropriate drug selection	5	2.1
Contraindicated	4	1.7
Necessary patient information not provided	4	1.7
**Drug with no indication**	**11**	**4.6**
No indication for drug	11	4.5

There was a significant relationship between the number of comorbidities and number of DRPs reported (*p*-value = 0.005), which consequently shows a positive relationship between polypharmacy and DRPs.

### Classes of drugs involved in drug-related problems

Through identifying DRPs according to the PCNE classification, 517 individual drugs were identified as causing a DRP. These drugs can be classified based on the Anatomical-Therapeutic-Classification (ATC) according to the organ system on which they act. Drugs acting on the central nervous system contributed to the most DRPs (136; 26.3%). The anti-infective classification also contributed to DRPs with (130; 25.2%) drugs. The frequency and percentage of drug groups causing DRPs are shown in Table 3.

**TABLE 3:** Classes and number of drugs involved in drug-related problems.

ATC code	Class	DRP (incident)	Responsible drug
N	Central nervous system; 136 (26.3%)	1. Failure to receive therapy (missed doses) – (64)	Paracetamol Tramadol Risperidone
		2. Therapeutic duplication (3)	Levetiracetam Sodium Valproate
		3. Dose too high (2)	
J	General anti-infective for systemic use; 130 (25.2%)	1. Drug regimen problem (5)	Rifafour^®^ [Rifampicin, Isoniazid, Pyrazinamide and Ethambutol]
		2. Drug interaction (3)	Dolutegravir interacting with Rifampicin
		3. Inappropriate dosage form (3)	Amoxicillin-clavulanic acid
		4. Dose too low (3)	Dolutegravir
		5. Inappropriate drug choice (intermediate culture sensitivity) (2)	Piperacillin-tazobactam
A	Alimentary tract and metabolism; 119 (23%)	1. Drug with no indication (5)	Lansoprazole
		2. Inappropriate therapy length (Stat dose given for too long) – (2)	Loperamide
C	Cardiovascular system; 50 (9.7%)	1. Drug indicated but not administered (2)	Furosemide
		2. Dose too high (2)	Enalapril
B	Blood and blood forming organs; 42 (8.1%)	1. Drug interaction (3)	Enoxaparin interacting with spironolactone and amlodipine
R	Respiratory system; 12 (2.3%)	1. Missed doses (10)	AB Nebs (formoterol and ipratropium bromide)
		2. Date omitted (2)	Fondaparinux sodium, Budesonide and formoterol fumarate dehydrate
H	Systemic hormonal preparations; 12 (2.3%)	1. Missed doses (6)	Hydrocortisone Dexamethasone
		2. Therapeutic duplication (4)	Prednisone
		3. No route of administration (1)	
		4. Dose too high (1)	
M	Musculo-skeletal system; 10 (1.9%)	1. Missed doses (6)	Allopurinol
		2. Date omitted (2)	Baclofen
		3. Drug with no indication (1)	Diclofenac
		4. Drug interaction (1)	
D	Dermatological; 4 (0.8%)	1. Missed doses (4)	Aqueous cream
			Emulsifying ointment
L	Antineoplastics and Immunomodulating agents; 2 (0.4%)	1. Missed doses (2)	Mesna

DRP, drug-related problem; ATC, anatomical-therapeutic-classification.

### Clinical Pharmacist’s recommendations

The clinical pharmacist made 277 recommendations to address DRPs across *n* = 181 patient file reviews. Nurses accepted 37.2% of the recommendations, while doctors accepted 22.7%. Of the recommendations, 78 (28.2%) were directed at nurses to correct administration errors, mainly through reminders and follow-ups. The most frequent recommendation to prescribers was to add a missing agent (58; 20.9%), communicated both verbally and in writing. Prescribers most often accepted recommendations to discontinue an agent (17; 51.5%), request cultures (9; 23.7%) or the addition of an agent where indicated (13; 22.4%). However, acceptance was low for switching agents (1; 11.1%) and dose adjustments (1; 10%). Table 4 summarises the acceptance rates of recommendations among nurses, doctors and patients. Prescription errors in Table 4 represent missing drug information such as start date, dose, dosing interval, in some cases patient weight and start time.

**TABLE 4:** Type of recommendations and acceptance rate.

Recommendation directed to:	Type of recommendation	Number (total = 277)		Number of interventions accepted		Number of interventions not accepted	
		*n*	%	*n*	%	*n*	%
Nurse	Recommended ensuring medications are given	78	28.2	29	37.2	49	62.8
Doctor	Recommended adding agent	58	20.9	13	22.4	45	77.6
	Recommended doing cultures	38	13.7	9	23.7	29	76.3
	Recommended correcting prescription error	33	11.9	0	0	33	100
	Recommended stopping an agent	33	11.9	17	51.5	16	48.5
	Recommended adjust dosing	10	3.6	1	10	9	90
	Recommended switching an agent	9	3.2	1	11.1	8	88.9
Patient	Counsel patient on drug therapy	18	6.5	18	6.5	0	0

## Discussion

This descriptive, quantitative study included *n* = 181 patient file reviews with the aim to identify and classify DRPs and to show the role clinical pharmacists can play as part of the multidisciplinary healthcare team to minimise DRPs.

**FIGURE 1-A1:**
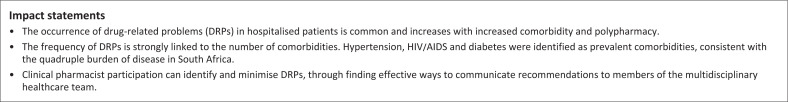
Impact statements highlighted by the research.

Patient files for *n* = 181 patients who met the inclusion criteria were reviewed. Two-thirds of the participants were male, with a median age of 44 years. This is contrary to the country’s demographics of 48% males with a median age of 28 years (Statisticstimes.com – South Africa demographics 2024). Comorbidities identified in the population were mostly hypertension, HIV/AIDS and diabetes, which is consistent with the quadruple burden of disease identified in South Africa (WHO 2018) - Refer to Appendix 1: Figure 1-A1. Most of the patients (especially the age group 41–60 years) had two or more comorbidities and were prescribed polypharmacy regimens consisting of more than five medications. These patterns are consistent with global observations on polypharmacy in populations with multiple comorbidities, also described during the 50^th^ ESCP symposium (Dreischulte et al. 2022a). This also aligns with the findings of Farrell, Szeto and Shamji (2011), who found that polypharmacy is common among older adults, with patients receiving 8 to 13 prescribed medications and experiencing an average of 2 to 3 DRPs. One of the assumed reasons for this trend in this study was complex comorbidities seen in this group of patients (Farrell et al. 2011).

Notwithstanding the fact that polypharmacy was present in more than 60% of the study population, no statistical significance (*p* = 0.089) was identified between polypharmacy and DRPs. This diverges from findings that found polypharmacy as the cause of high DRP prevalence, even in other African countries (Buda et al. 2021; Mamo & Alemu 2020; Mekonnen, Ayalew & Tegegn 2021). However, the study still found a high incidence of DRPs (241 with an average of 1.76 per patient). Studies performed in internal medicine wards in both developed and developing countries have shown similar results of between 1.3 and 2.6 DRPs per patient (Adhikari et al. 2021; Guignard et al. 2015; Pokkuluri et al. 2023). Additionally, the majority of these DRPs are in the age group of 41–60 years (Pokkuluri et al. 2023; Shareef, Sandeep & Shastry 2014). It was further speculated that increased DRPs may be the result of increased comorbidities and polypharmacy (Pokkuluri et al. 2023). The influence of comorbidities and polypharmacy on DRPs was demonstrated by a study conducted in the elderly that showed that elderly patients had a higher chance of developing DRPs because of the high number of comorbidities and drugs prescribed in this population (Hailu et al. 2020).

In this study, which was carried out in a developing country, failure to receive therapy (under-administration or drug not administered at all) was the highest reported DRP, which does not correlate to polypharmacy at all but rather with systemic challenges. In developing countries, non-administration of medication was cited for around 21% of DRPs (Abunahlah et al. 2018; Leite et al. 2016). This is in contrast with developed countries who reported drug–drug interactions (35% – 48%) as the largest contributor to DRPs (Lenssen et al. 2016; Mohammed et al. 2017; Pokkuluri et al. 2023). Factors such as shortage of nursing staff, high workload, few clinical pharmacists, limited resources and a weak medication dispensing system are some contributors identified to lead to incidences of non-administration of medication (Abrahams, Thani & Kahn 2022; Keers et al. 2018; Leite et al. 2016). Similarly, the South African Nursing Council (SANC) recognises that the ratio of nurses to patients indicates short staffing at 1:213 in South Africa (Abrahams et al. 2022).

Furthermore, drug identification using the ATC classification system showed that central nervous system drugs were most frequently involved in DRPs, accounting for 136 cases (26.3%). Paracetamol was most frequently linked to missed doses, therapeutic duplication and overdose – likely because of its widespread use for pain management, which may lead to overprescription. The practice of prescribing paracetamol ‘as needed, or PRN’ was not regarded as a missed dose. A similar approach was taken in cases where a nurse indicated on the prescription that the patient refused medication or was vomiting in the context of oral dosage formulations. Results from other studies reported on paracetamol in terms of overdose or therapeutic duplication, rather than missed doses (Guignard et al. 2015). Although anti-infectives were not the highest contributors to DRPs in this study, the number of DRPs caused by these drugs was 130 (25.2%), making it the second highest drugs to contribute to DRPs. In recent studies, the anti-infective for systemic use, especially antimicrobials, were reported as the class that contributed the most to DRPs, especially because of dosing (over or underdosing) and treatment duration issues (Adhikari et al. 2021; Pokkuluri et al. 2023).

In this study, the clinical pharmacist used the PCNE system to identify and classify DRPs and made 277 recommendations to other members of the multidisciplinary healthcare team. To address missed medication doses, the clinical pharmacist reminded nurses to ensure all medications were administered as prescribed. Most recommendations to prescribers involved adding omitted treatments, particularly antiretroviral or antituberculosis drugs.

Recommendations to discontinue a drug or request culture and sensitivity tests were more likely to be accepted by the doctor. The method of communicating the recommendation may have influenced the acceptance rate of recommendations. When direct verbal communication was used to make recommendations, the acceptance rate was higher than when messages were written in the patient’s clinical notes, where prescribers were not available for communication. Evidence for successful verbal communication was shown by pharmacotherapy experts, in a study where they participated in medical rounds with prescribers, communicating directly to achieve an acceptance rate of 84% (Guignard et al. 2015). In several studies, the presence of clinical pharmacists at ward level has resulted in positive change by reducing DRPs, which is observed by other healthcare professionals (Ahmed et al. 2021; Chevalier et al. 2016; Movva et al. 2015; Pokkuluri et al. 2023).

### Recommendations

The findings point to the importance of conducting further research on the underlying factors associated with DRPs. This may expose a deeper problem than initially expected, especially how to reduce missed medication doses because of the high workload and staff shortages. These findings could support the creation of systems to prevent DRPs. With a clinical pharmacist present in the ward, ways to improve communication can be explored to increase the acceptance of recommendations.

## Conclusion

The study highlighted the clinical pharmacist’s capability to recognise and classify DRPs in hospitalised patients. The DRPs in this study ranged in severity from omitted doses to therapeutic duplication and drug–drug interactions. The incidence of DRPs increased with higher comorbidities and age. The inclusion of a clinical pharmacist within the multidisciplinary team could play a significant role in reducing the incidence of DRPs in hospitalised patients.
